# Development of New Polymorphic Microsatellite Loci for the Barley Stem Gall Midge, *Mayetiola hordei* (Diptera: Cecidomyiidae) from an Enriched Library

**DOI:** 10.3390/ijms131114446

**Published:** 2012-11-08

**Authors:** Maha Mezghani-Khemakhem, Dhia Bouktila, Nathalie Casse, Houcine Maaroufi, Mohamed Makni, Hanem Makni

**Affiliations:** 1 UR11ES10 Genomics of Crop Insect Pests, Faculté des Sciences de Tunis, University of Tunis El-Manar, El Manar 2092, Tunisia; E-Mails: dhia_bouktila2000@yahoo.fr (D.B.); maaroufi.houcine@gmail.com (H.M.); md.makni@fst.rnu.tn (M.M.); hanem.makni@isajc.rnu.tn (H.M.); 2 Higher Institute of Biotechnology of Béja, University of Jendouba, Avenue Habib Bourguiba, Béja 9000, Tunisia; 3 Laboratory Mer, Molécules, Santé (MMS), UFR Sciences and Techniques Le Mans, University of Maine, Le Mans 72085, France; E-Mail: nathalie.casse@univ-lemans.fr; 4 Higher Institute of Animation for Youth and Culture, University of Tunis, Bir El Bey 2055, Tunisia

**Keywords:** *Mayetiola destructor*, dinucleotide, trinucleotide, molecular ecology

## Abstract

Using an enriched library method, seven polymorphic microsatellite loci were isolated from the barley stem gall midge, *Mayetiola hordei*. Polymorphism at loci was surveyed on 57 individual midges collected on barley in Tunisia. Across loci, polymorphism ranged from two to six alleles per locus. The observed heterozygosity varied between 0.070 and 0.877. Based on the number of alleles detected and the associated levels of heterozygosity, we believe that these loci will prove useful for population genetic studies on *M. hordei*.

## 1. Introduction

In North Africa, the Hessian fly, *Mayetiola destructor* (Say) and the barley stem gall midge, *M. hordei* (Keiffer) are two sympatric sibling species. In Morocco, wheat is exclusively colonized by *M. destructor* and displays no galls, whereas barley is exclusively infested by *M. hordei* that produces stem galls [1]. In Tunisia, the situation is more complex as there is no strict association between the cereal species and the *Mayetiola* species. In fact, barley is mainly infested by *M. hordei* which produces galls; but wheat could be infested by both *Mayetiola* species without gall producing [2].

Nowadays, the most economic and practical means of controlling *Mayetiola* sp. midges is the use of resistant cultivars. In *M. destructor*, a highly specific gene-for-gene interaction was demonstrated, which involves dominant resistance genes (*H*) in the host plant, and recessive avirulence genes (*avr*) in the insect [3]. As a result, sixteen *M. destructor* biotypes [4] have been identified on the basis of their differential response (virulence or avirulence) to four common wheat cultivars carrying the *H3*, *H5*, *H6* or *H7H8* genes. Virulent biotypes occur in nature as a result of selection pressure exerted by resistant host plant genotypes. However, in USA, Hessian fly virulence has been found to some resistance genes that have not been deployed in wheat cultivars [5]. Unfortunately, it is not yet known, whether virulent biotypes exist in *M. hordei*[6]. Therefore, studying the genetic variability of Tunisian *M. hordei* populations on barley and wheat is most probably a key step towards a clear understanding of the genetic patterns of interaction associating *M. hordei* to wheat and/or barley. Previous studies using microsatellite markers have shown that host plant species is a major selective factor acting on the genetic structure of *M. hordei* as the majority of the microsatellites loci were monomorphic for individuals collected on barley and polymorphic for those collected on wheat [7]. Hence, in order to have a deeper comprehension of the genetic organization of *M. hordei* populations on barley, in the present study, we develop additional microsatellite loci for *M. hordei*, using an enriched microsatellite library protocol. Combined with loci previously isolated in the barley stem gall midge, these new microsatellite markers provide valuable tools to study population genetics of this species.

## 2. Results and Discussion

A total of 15 loci were originally isolated, but only seven of them were suitable and were subsequently used for polymorphism screening. Levels of polymorphism were tested on 57 individuals of *M. hordei* collected on barley from several geographical locations. Number of alleles per locus ranged from two to six and the observed heterozygosity varied from 0.070 to 0.877 (Table 1). Deviation from Hardy-Weinberg equilibrium (HWE) and linkage disequilibrium were calculated using the GENEPOP software version 3.4 [8]. Three loci, MhA6, MhC2-1 and MhC3, did not show deviations in the proportion of heterozygous from that expected under the HWE. Two loci (MhB2 and MhF11) showed a significant deficit of heterozygous and the two remaining loci MhA10 and MhC1-1 showed an excess of heterozygous, with *p* < 0.001. To test for genotyping errors and aid in identification of possible null alleles, loci were further analyzed in MircroChecker Software [9]. Results showed that five loci had no null allele in any of the samples, and two (MhB2 and MhF11) had potential null alleles (Table 1). For these two loci, redesign of primers would produce consistent amplification and could eliminate the null allele. Tests for linkage disequilibrium showed no significant linkage between pairs of loci after Bonferroni’s correction.

## 3. Experimental Section

Total genomic DNA was extracted from a bulk of 200 *M. hordei* individuals, using the CTAB method [10]. The DNA was digested with *RsaI* (Promega) for 1H at 37 °C, then, ligated into adaptators and PCR-amplified. PCR products were subsequently hybridized with three biotinylated microsatellite probes, corresponding to motifs (CAA)_8_, (TG)_12_ and (AG)_12_. The tagged sequences were captured using streptavidin**-**coated magnetic beads, then PCR-amplified using SuperSNX24 Forward: 5′-GTTTAAGGCCTAGCTAGCAGAATC-3′ and SuperSNX24+4P Reverse: 5′-pGATTCT GCTAGCTAGGCCTTAAACAAAA-3′ adaptator sequences as primers [11]. Final microsatellite-rich products were inserted into the pGEM-T vector (Promega) and transformed into DH5α (NEB) competent cells according to the standard protocol. Libraries were stored at −80 °C. To isolate colonies for sequencing, cells from the glycerol stock were spread on X-gal/IPTG/ampicillin-LB agar plates. One hundred and two (102) recombinant clones were obtained. Screening these clones using T7 and SP6 PCR primers enabled the selection of 62 clones harbouring inserts varying in length between 500 bp and 1300 bp. These were purified using the QIA prep plasmid DNA prep Kit (QIAGEN, Duesseldorf, Germany) and sent for sequencing (Genome Express). Thirteen sequences containing 15 microsatellite loci were obtained and deposited in GenBank under accession numbers JN585338 to JN585350. The two sequences JN585343 and JN585344 contained two microsatellite loci each.

Locus specific PCR primers were designed using the primer3 Software [12]. Variability at each microsatellite locus was analysed using 57 *M. hordei* individuals collected at the pupal stage then reared until emergence. Sampling was done on barley from seven sites located in the north (Béjà and Kélibia), center (Kairouan) and south (Sfax and Gabès) of Tunisia. Microsatellite PCR reactions were carried out in a final volume of 25 μL containing 20 ng of DNA, 1.5 μL 10× buffer, 100 μM dNTPs, 0.5 μM of each primer and 0.25 U Taq DNA polymerase (QBiogene). The cycling conditions on “MJ Research, INC” thermocycler were as follows: initial denaturation at 94 °C for 5 min followed by 40 cycles of 95 °C for 30 s, annealing temperature of each locus for 30 s and extension at 72 °C for 1 min, with a final extension at 72 °C for 10 min. PCR products were separated on 8% polyacrylamide denaturing gels and visualised under UV lights after ethidium bromide staining. The microsatellite alleles were sized by reference to the sequenced allele and to a molecular weight ladder, the marker 10 (Euromedex, Strasbourg, France).

## 4. Conclusions

The effective management of the barley stem gall midges requires an understanding of the population structure of this species. This study describes seven microsatellite loci which are likely to be used in combination with loci previously isolated in *M. hordei* to understand biotype adaptation of this crop pest. Furthermore, the pairs of primers described here may be suitable for assessment of genetic diversity and population structure of the wheat pest *M. destructor.*

## Figures and Tables

**Table 1:** Primer sequences and characteristics of seven microsatellite loci from *Mayetiola hordei.* Observed heterozygosity (*H*_O_), expected heterozygosity (*H*_E_) and the *p*-value, probability of HW exact tests, are given.

Locus Name	Repeat motif	Primer sequence (5′→3′)	*T*_a_ (°C)	No. of alleles	Size range	*H*_O_	*H*_E_	*p*-Values	GenBank accession no.	Null allele frequency estimate
MhA6	(CAAAAA)_4_	MHA6F: AATTATGTAAACCGAACCGAAC MHA6R: CGAATCCAAAGAGGAAGTGG	54	6	177–185	0.5789	0.6122	0.0976	JN585338	14
MhA10	(CAA)_19_	MHA10F: CGTCGCAATCATTTCTTTCA MHA10R: TGCATTTGAGCCACATTTTC	56	5	176–185	0.6667	0.5314	0.0017	JN585339	−1346
MhB2	(CA)_13_	MHB2F: TGGGCATAATTTTTCCGAAT MHB2R: TTCCAAAAACAGTTCGTTCAA	54	6	149–159	0.1228	0.6850	0.0000	JN585341	3961
MhC1-1	(TG)_26_	MHC1-1F: TGTGGGCTCAAATGGAAAAT MHC1-1R: GAAGGTTTCCAGTCGCACAC	56	5	134–140	0.8772	0.6711	0.0000	JN585343	−1741
MhC2-1	(GTT)_8_	MHC2-1F: TGTTGAATCCTATAACAATTTCATGTT MHC2-1R: TCATAATCCGGGGCAATAAA	56	2	121–124	0.2456	0.2155	0.5830	JN585344	−1314
MhC3	(TG)_16_	MHC3F: CGTCGCAATCATTTCTTTCA MHC3R: TGCATCTGAGCCACATCTTC	56	2	177–179	0.0702	0.0997	0.1292	JN585345	784
MhF11	(CT)_10_	MHF11F: TCCATATCCACATACGGATTCA MHF11R: TTTGCTGTTGTTGGAAGCAG	56	4	141–145	0.2632	0.5339	0.0000	JN585350	2273
